# Perceived importance of and attitude towards physical education in Austrian adolescents – The role of sex, age and weight status

**DOI:** 10.3934/publichealth.2025028

**Published:** 2025-05-07

**Authors:** Clemens Drenowatz, Gerson Ferrari, Carla Greier, Gerhard Ruedl, Klaus Greier

**Affiliations:** 1 Division of Sport, Physical Activity and Health, University of Education Upper Austria, Linz, Austria; 2 Escuela de Ciencias de la Actividad Física, el Deporte y la Salud, Universidad de Santiago de Chile (USACH), Santiago, Chile; 3 Faculty of Health Sciences, Universidad Autónoma de Chile, Providencia, Chile; 4 Department of Sports Science, Leopold-Franzens University Innsbruck, Innsbruck, Austria

**Keywords:** school sports, physical activity, high school, middle school, youth, physical activity enjoyment, body weight

## Abstract

Physical education (PE) is often considered an ideal setting for the promotion of physical activity (PA) among adolescents and for the encouragement of an active lifestyle in adulthood. In order to achieve this goal, however, it is necessary to foster a positive attitude towards PE. The present study examined attitudes towards and the perceived importance of PE among Austrian adolescents. A total of 3011 adolescents (47.5% female) between 10 and 17 years of age completed a standardized questionnaire during regular school hours. In addition, body weight and height were measured. Overall, 78.5% stated that PE is important to them and 84.3% believed that PE positively affects their health and well-being. PE also motivated 68.4% of the participants to engage in leisure time PA, and 69.9% would like to have daily PE. Further, 91.0% reported a good relationship with their PE teacher while 68.6% considered themselves to be good at sports. Nevertheless, there was a significant decline in the perceived value of PE with increasing age, and among girls, as well as those with excess body weight, perceived PE to be less important. As these groups are particularly vulnerable for low PA levels, it is important to provide challenging and enjoyable experiences that foster a positive attitude towards PE. Ensuring positive experiences throughout students' school careers could play a key role in promoting an active lifestyle later in life.

## 1. Introduction

Regular physical activity (PA) is a key determinant of both physical and psychosocial health. In children and adolescents, PA has been associated with a healthy body weight, cardiometabolic health, bone strength, physical fitness as well as academic performance and overall quality of life [1],[2]. Current PA guidelines recommend at least 60 minutes per day of moderate to vigorous PA for children and adolescents, including at least 3 days per week with vigorous PA and muscle and bone strengthening activities [1],[3]. Only 20% of adolescents, however, meet these recommendations [4], while engagement in sedentary activities (e.g., computer games, television use, etc.) has been increasing [5],[6]. This shift in behavioral choices has significant implications for public health as insufficient PA among adolescents is considered a leading risk factor for excess body weight, non-communicable diseases, and mental health disorders, which may lead to chronic health problems in adulthood [7]–[9]. As a result, the global healthcare costs of physical inactivity are estimated to exceed $300 billion by 2030 [10].

Adolescence provides a critical window of opportunity for the promotion of an active and healthy lifestyle. Research has shown that school-based PA contributes to higher PA levels among youth and later in life [11],[12]. Physical education (PE), which refers to the structured implementation of PA within the school context, is considered one of the most cost-effective strategies for promoting PA in youth and the next generation of adults [13],[14]. As PE is part of the curriculum in many countries [15], it can reach a majority of adolescents irrespective of socio-economic background. In fact, it has been argued that for many children and adolescents, school represents the only opportunity for regular PA [16]. Furthermore, schools have been estimated to be the primary source of PA for 80% of adolescents, which has been attributed to parental concerns about safety during free play and economic pressures that may limit access to club sports [17]. PE provides an opportunity for structured PA that includes a variety of activities, such as team and individual games and sports, as well as dance, and is usually led by qualified and accountable teachers in order to promote a healthy and active lifestyle [15]. In addition, PE plays an important role in the development of physical fitness and well-being and contributes to the psychological and social development and physical health of adolescents [2],[18],[19]. As the primary institutional setting for motor development and PA in children and adolescents [2], PE plays a crucial role in building fundamental movement skills, which are essential for lifelong PA and sports participation. Notably, a lack of PE exposure in school has been linked to an increased risk of physical inactivity in adulthood [20]–[22].

In order to experience the previously mentioned benefits, sustained active engagement in PE is necessary. Accordingly, a positive attitude towards PE appears to be an important contributor for PE participation, which has been shown to positively affect leisure time PA as well [23]–[26]. In fact, intervention programs targeting attitude towards PA have been shown to increase time spent in moderate-to-vigorous PA (MVPA) during PE [23]. Even though PE is one of the most popular subjects in schools [27],[28], there are individual differences in the perception and benefits of PE. For example, PE was valued more favorably among males and those of younger age [27],[29],[30]. Additionally, more physically active youth and those with higher skill levels generally rate PE more positively than their less-skilled peers [31]. Results on the association between weight status and attitudes towards PE, on the other hand, have been equivocal [28],[29]. These studies, however, were conducted prior to the implementation of COVID-19 restrictions, which had a significant impact on teaching modes, PA, and physical fitness [32]–[35]. Given the important role in the promotion of an active and healthy lifestyle, further research on correlates of PE engagement is needed. The present study, therefore, examined attitudes towards and perceived importance of PE among 10- to 17-year-old secondary school students and the association with sex, age, and weight status.

## 2. Materials and methods

Out of 10 public schools that combine middle and high school (grades 5 to 12) in the Federal State of Tyrol, 5 schools (50% of available schools) were selected via a random number generator. In order to obtain a representative sample, a similar number of middle schools (grades 5 to 8) were randomly selected as well. Elementary schools were not invited to participate, as PE in grades 1 through 4 is taught by the regular classroom teacher, rather than a specialized PE teacher.

In Austrian secondary schools, there are 3 class periods (50 minutes per period) of mandatory PE per week in grades 5 through 8 and 2 class periods (50 minutes per period) of mandatory PE per week in higher grades, which are taught by college-educated, specialized PE teachers. The standardized curriculum consists of a variety of activities, including ball games, gymnastics, swimming, and winter sports, as well as strength and conditioning. In addition to the development of motor competence and an understanding of basic training principles, social and self-competence are emphasized. Given the variety of activities, teachers have some flexibility on the specific content of the class and how key competencies are addressed.

All schools that were contacted agreed to participate in the study and the study protocol was approved by the University of Innsbruck Institutional Review Board. Parents or legal guardians provided written informed consent prior to data collection, and participants provided oral assent at the time of data collection. A total of 3011 adolescents (47.5% female) with an average age of 13.2 ± 2.1 years completed the questionnaire. To assess potential changes in the attitude towards PE throughout adolescence, participants were categorized into 4 age groups: 10–11 years, 12–13 years, 14–15 years, and >15 years.

Attitudes towards and perceived importance of PE were assessed using a standardized questionnaire, which has been used previously [27]. The questionnaire was developed by experts and was tested prior to its initial implementation to ensure that the included statements were understandable for the target age group. Data collection occurred between April and June 2024 at the beginning of a regular PE class between 9:00 am and 12:00 pm in the schools' gymnasium. The questionnaire was administered by a researcher in a quiet atmosphere, and completion took between 5 and 10 minutes. Specifically, participants rated their agreement with 6 statements regarding the perceived importance of PE and its effect on health and well-being, motivation towards PE, their relationship with the PE teacher, and their perceived competence in PE using a four-point Likert scale ranging from strongly disagree to completely agree. In addition, they were asked to evaluate the importance of PE in relation to other subjects using 3 answer options (less important, equally important, more important). Answers were converted to numeric data with a value of 1 indicating strongly disagree or less important and a value of 4 indicating completely agree or more important.

Prior to completing the questionnaire, body height and weight were measured according to standard procedures, with participants wearing sports clothes and no shoes. Body height was measured to the nearest 0.1 cm using a mobile stadiometer (SECA® 217, Seca; Germany), and body weight was measured to the nearest 0.1 kg using a calibrated scale (SECA® 803, Seca; Germany). Subsequently, body mass index (BMI, kg/m²) was calculated and converted to BMI percentiles (BMIPCT) using German reference values [36]. BMIPCT were used to classify participants as underweight (BMIPCT < 10), normal weight (10 ≤ BMIPCT ≤ 90) or overweight/obese (BMIPCT > 90).

### 2.1. Statistical analysis

Descriptive statistics are reported as means with standard deviations for continuous data, while prevalence rates are reported for categorical data. Sex differences in anthropometric characteristics were examined via *ANOVA* and Mann-Whitney *U* tests were used to examine differences in attitudes towards and perceived importance of PE. Differences across weight status and age categories were examined via Kruskal-Wallis tests separately for boys and girls. Statistical analyses were performed with SPSS 29.0 (Armonk, NY, USA) with the significance set at *p* < 0.05 and Bonferroni adjustment was applied for multiple comparisons.

## 3. Results

A total of 1429 female and 1582 male participants between 10 and 17 years of age provided complete data. Across the entire sample, 31.0% were in the 12–13-year age group, 26.5% were in the 10–11-year group, and 23.9% were in the 14–15-year group. The remaining participants (18.6%) were 15 years or older. Almost three quarters (73.2%) of the participants were normal weight, 17.7% were considered as overweight/obese, and 9.1% were considered as underweight. There were no sex differences in age, but boys were significantly taller and heavier than girls (Table 1). Boys also had higher BMI percentiles than girls, but there were no significant sex differences across weight categories.

**Table 1.** Anthropometric characteristics of the total sample and separately for male and female participants (Values are mean with standard deviation and prevalence for weight status).

**Project**	**Total sample** ***n* = 3011**	**Female only** ***n* = 1429**	**Male only** ***n* = 1582**
Age (years)	13.2 ± 2.1	13.1 ± 2.1	13.2 ± 2.1
Height (cm)*	160.8 ± 12.3	158.6 ± 10.1	162.7 ± 13.8
Body Weight (kg)*	52.4 ± 14.7	50.9 ± 13.5	53.8 ± 15.6
BMI Percentile*	53.7 ± 29.8	52.0 ± 30.3	55.1 ± 29.2
Normal Weight (%)	73.2	72.6	73.8
Overweight/Obese (%)	17.7	17.1	18.3
Underweight (%)	9.1	10.3	8.0

Note: * sig. sex difference, *p* < 0.01.

The majority of adolescents (78.5%) mostly or completely agreed that PE was important to them, and 84.3% reported that PE positively affected their health and well-being. PE also motivated more than two thirds of the participants (68.4%) to engage in exercise or PA during leisure time. A total of 68.6% considered themselves to be good at sports, and 91.0% had a good relationship with their PE teacher. There was also a large preference for daily PE (69.9%) even though less than half of the participants (41.9%) considered PE to be more important than other subjects (Table 2). While both male and female participants displayed a positive attitude towards PE with more than 50% agreeing mostly or completely with the respective statements, the agreement rate was higher in boys compared to girls (*p* < 0.01).

**Table 2.** Attitude towards physical education in the total sample and separately for female and male participants [Values are prevalences (%)].

*Project*		*Completely Agree*	*Mostly Agree*	*Mostly Disagree*	*Completely Disagree*
PE is important to me.*	**TOTAL**	**43.0**	**35.5**	**17.5**	**4.0**
	FEMALE	35.8	38.9	20.6	4.7
	MALE	49.5	32.4	14.7	3.4
PE positively affects my health and well-being.*	**TOTAL**	**43.8**	**40.5**	**13.5**	**6.6**
	FEMALE	36.0	45.0	16.6	2.4
	MALE	50.8	36.3	10.6	2.2
PE motivates me to engage in exercise/PA during leisure time.*	**TOTAL**	**30.9**	**37.5**	**25.0**	**6.6**
	FEMALE	20.2	40.9	33.1	5.9
	MALE	40.6	34.5	17.7	7.3
I would like to have PE daily.*	**TOTAL**	**41.6**	**28.3**	**21.4**	**8.7**
	FEMALE	30.6	33.3	26.2	9.9
	MALE	51.5	23.8	17.1	7.6
I have a good relationship with my PE teacher.*	**TOTAL**	**54.9**	**36.1**	**7.6**	**1.4**
	FEMALE	47.8	40.4	10.0	1.7
	MALE	61.4	32.1	5.4	1.1
I consider myself to be good in sports.*	**TOTAL**	**30.9**	**37.7**	**25.6**	**5.8**
	FEMALE	22.0	36.8	33.3	7.8
	MALE	38.8	38.5	18.6	4.0

		*More Important*	*Equally Important*	*Less mportant*

Importance of PE compared to other subjects.*	**TOTAL**	**41.9**	**41.7**	**16.3**
	FEMALE	35.4	44.6	19.9
	MALE	47.9	39.1	13.1

Note: * sig. sex difference, *p* < 0.01.

**Table 3.** Attitude towards physical education by age group separately for female and male participants [Values are prevalences (%)].

***Project***		***Completely Agree***		***Mostly Agree***		***Mostly Disagree***		***Completely Disagree***	

		Girls	Boys	Girls	Boys	Girls	Boys	Girls	Boys
**PE is important to me.**	10–11 yrs	64.3	70.5	27.3	24.8	7.6	4.2	0.8	0.5
	12–13 yrs	38.7	56.6	51.2	34.0	9.5	7.3	0.7	2.1
	14–15 yrs	17.8	37.8	43.7	36.5	32.9	20.6	5.5	5.1
	>15 yrs	9.1	25.7	28.7	34.5	45.3	32.6	16.9	7.2
**PE positively affects my health and well-being.^1,2,a^**	10–11 yrs	53.2	66.6	34.9	27.0	9.6	4.7	2.3	1.7
	12–13 yrs	45.1	62.7	43.7	29.1	10.1	6.7	1.1	1.5
	14–15 yrs	19.7	43.9	53.8	41.4	21.5	12.9	4.9	1.8
	>15 yrs	13.8	20.5	51.6	53.4	32.7	21.5	2.0	4.6
**PE motivates me to engage in exercise/PA during leisure time.^1,a^**	10–11 yrs	31.6	51.5	42.0	32.7	20.8	12.9	5.6	3.0
	12–13 yrs	24.8	45.7	43.5	37.3	27.7	13.6	4.0	3.4
	14–15 yrs	11.1	34.3	41.8	36.0	40.3	19.3	6.8	10.4
	>15 yrs	5.5	26.4	33.1	30.3	52.8	28.3	8.7	15.0
**I would like to have PE daily.**	10–11 yrs	54.4	71.3	32.9	21.0	7.8	4.5	4.8	3.2
	12–13 yrs	35.6	54.1	39.8	26.2	20.7	14.5	4.0	5.2
	14–15 yrs	12.0	44.9	34.8	22.1	41.2	25.6	12.0	7.4
	>15 yrs	8.3	30.0	20.5	26.1	45.7	26.7	25.6	17.3
**I have a good relationship with my PE teacher.^1,2,c^**	10–11 yrs	62.3	66.8	26.1	25.2	8.4	7.2	3.3	0.7
	12–13 yrs	50.3	61.8	41.1	31.2	7.9	5.2	0.7	1.7
	14–15 yrs	39.1	63.5	49.2	32.0	10.8	3.3	0.9	1.3
	>15 yrs	31.9	50.8	50.4	42.7	15.4	5.9	2.4	0.7
**I consider myself to be good in sports.^a^**	10–11 yrs	33.4	48.0	44.6	36.1	19.0	13.9	3.0	2.0
	12–13 yrs	27.5	44.0	37.6	36.9	31.2	14.9	3.7	4.2
	14–15 yrs	10.8	34.5	35.7	40.9	45.2	19.4	8.3	5.1
	>15 yrs	9.1	24.1	24.8	41.0	44.1	29.6	22.0	5.2

		***More Important***		***Equally Important***		***Less Important***	

**Importance of PE compared to other subjects.^1^**	10–11 yrs	52.2	65.6	41.3	29.7	6.6	4.7
	12–13 yrs	44.0	52.0	46.6	39.2	9.5	8.8
	14–15 yrs	20.0	43.7	48.0	39.1	32.0	17.3
	>15 yrs	13.8	23.5	42.1	51.1	44.1	25.4

Note: sig. differences across all age groups except for: ^1/a^ no difference between 10–11 years and 12–13 years in girls (1) or boys (a); ^2/b^ no difference between 14–15 years and >15 years in girls (2) or boys (b); ^c^ only >15 years sig. different from other age groups in boys.

Attitudes towards PE by age:

There was a significant difference in the attitude towards and perceived importance of PE across age groups in girls and boys (*p* < 0.01). Specifically, the perceived importance of PE and the preference for daily PE differed significantly across all age groups in both girls and boys (*p* < 0.01) (Table 3). Among girls, there was also a significant difference between all age groups concerning their perceived sports abilities, while there was no difference between the younger age groups in boys. Further, there was no difference in the perceived positive effects of PE on health and well-being among boys between 10 and 13 years of age. The motivation of PE for leisure time exercise/PA did not differ between the ages 10 and 13 among both girls and boys, while it significantly declined after the age of 13 (*p* < 0.01). Among girls there was a significant age difference for the perceived positive effects of PE on health and well-being and their relationship with the PE teacher between 10- to 13-year-olds and those 15 years and older. Similarly, the perceived importance of PE did not differ among 10- to 13-year-old girls, while there was a significant difference across all age groups in boys (*p* < 0.01).

**Figure 1.**
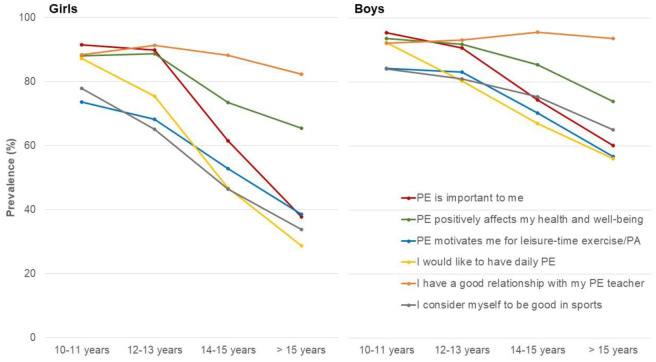
Level of agreement (completely and mostly agree) across different age groups for girls and boys [Values are prevalences (%)].

Overall, the perceived importance of PE, its impact on health and well-being, and the perception of their own abilities in sports declined significantly with increasing age in boys and girls (*p* < 0.01). There was also a significant decline, albeit at a high level, regarding a good relationship with their PE teacher among girls (*p* < 0.01), while no significant change was observed among boys (Figure 1). Girls further displayed a more pronounced decline in their attitude towards PE with less than 40% of girls above 15 years of age considering PE to be important or feeling that PE motivates them for engagement in exercise/PA during leisure time. Furthermore, less than 30% of girls above 15 years of age would like to have daily PE and only 33.9% considered themselves to be good at sports compared to prevalences above 70.0% and 69.0%, respectively, in the younger age groups. Among boys, on the other hand, the agreement with the respective statements remained above 50% across all age groups.

**Figure 2.**
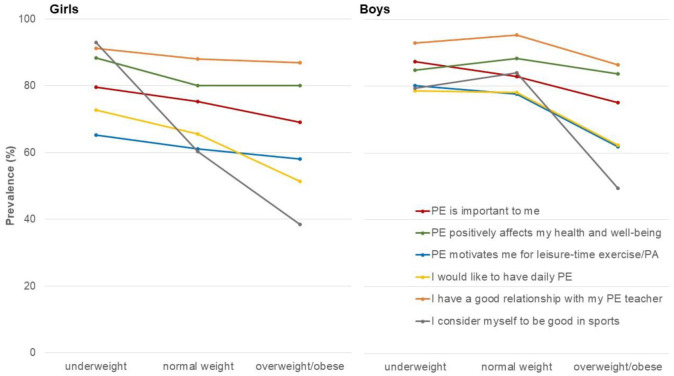
Level of agreement (completely and mostly agree) for underweight, normal weight and overweight/obese girls and boys [Values are prevalences (%)].

Attitude towards PE by weight status:

Both, male and female adolescents with overweight/obesity considered PE to be less important than their peers and were less likely support daily PE compared to their peers (*p* < 0.01) (Table 4). Boys with overweight/obesity displayed a lower perception of health benefits of PE (*p* = 0.04). In girls, on the other hand, underweight was associated with a higher health perception of PE (*p* = 0.04). There was no difference in the motivation of PE for active leisure time choices in girls, while boys with overweight/obesity were less likely to consider PE to be motivating for leisure time exercise/PA (*p* < 0.01). Adolescents with overweight/obesity also were less likely to consider themselves to be good at sports (*p* < 0.01), and they were less likely to report a good relationship with their PE teacher (*p* = 0.02 among girls and *p* = 0.04 among boys) (Figure 2).

**Table 4.** Attitude towards physical education by weight status separately for female and male participants [Values are prevalences (%)].

***Project***		***Completely Agree***		***Mostly Agree***		***Mostly Disagree***		***Completely Disagree***	

		Girls	Boys	Girls	Boys	Girls	Boys	Girls	Boys
**PE is important to me.^1,2,a,b^**	Underweight	42.9	60.3	36.7	27.0	17.7	10.3	2.7	2.4
	Normal weight	37.0	52.0	38.3	30.9	19.8	13.6	4.9	3.4
	Overweight/Obese	26.1	34.6	42.9	40.5	26.1	21.1	4.9	3.8
**PE positively affects my health and well-being.^2,3,a,b^**	Underweight	44.2	57.9	44.2	27.0	11.6	10.3	0.0	4.8
	Normal weight	36.7	52.1	43.4	36.2	16.9	10.0	3.0	1.7
	Overweight/Obese	27.8	42.6	52.2	41.2	18.4	13.1	1.6	3.1
**PE motivates me to engage in exercise/ PA during leisure time.^a^**	Underweight	24.5	38.9	40.8	41.3	25.9	11.1	8.8	8.7
	Normal weight	20.7	42.6	40.4	35.1	33.4	15.2	5.5	7.1
	Overweight/Obese	15.1	33.2	42.9	28.7	36.3	30.8	5.7	7.3
**I would like to have PE daily.^1,2,a,b^**	Underweight	41.5	54.8	31.3	23.8	17.0	13.5	10.2	7.9
	Normal weight	31.4	55.5	34.1	22.7	25.2	15.6	9.3	6.2
	Overweight/Obese	20.4	33.9	31.0	28.4	36.3	24.6	12.2	13.1
**I have a good relationship with my PE teacher.^1,a,b^**	Underweight	50.3	61.1	40.8	31.7	7.5	6.3	1.4	0.8
	Normal weight	49.6	64.3	38.6	31.0	10.1	3.7	1.7	1.0
	Overweight/Obese	38.8	49.8	48.2	36.7	11.0	11.8	2.0	1.7
**I consider myself to be good in sports.^1,2,3,a,b^**	Underweight	49.0	46.8	34.0	32.5	12.9	16.7	4.1	4.0
	Normal weight	21.6	44.1	38.7	39.8	34.1	13.4	5.6	2.7
	Overweight/Obese	7.8	13.8	30.6	35.6	42.0	40.8	19.6	9.7

		*More Important*		*Equally Important*		*Less Important*	

**Importance of PE compared to other subjects.^1,a^**	Underweight	34.0	48.4	49.0	37.3	17.0	14.3
	Normal weight	37.5	49.3	42.8	39.2	19.7	11.5
	Overweight/Obese	27.3	41.5	49.8	39.4	22.9	19.0

Note: ^1^ sig. difference between normal weight and overweight/obese girls; ^2^ sig. difference between underweight and overweight/obese girls; ^3^ sig. difference between underweight and normal weight girls; ^a^ sig. difference between normal weight and overweight/obese boys; ^b^ sig. difference between underweight and overweight/obese boys.

## 4. Discussion

Given the potential impact of PE in the promotion of an active lifestyle among adolescents and later in life, the present study examined the attitudes towards and perceived importance of PE among Austrian middle- and high-school students. The results indicated that adolescents generally rated PE positively, though there was a decline in the perceived value of PE as age increased. Boys displayed a more positive attitude towards PE compared to girls, and the decline in the positive attitude and perceived importance with increasing age was more pronounced among girls. In addition to sex and age, significant differences were observed across weight categories. Adolescents with overweight/obesity displayed a less positive attitude towards PE, although there was no difference in the perceived health benefits of PE. Participants with overweight/obesity also considered themselves less skilled in sports, but only boys reported that PE was less motivating for leisure time PA compared to their peers. Despite a generally good relationship with their PE teacher (>86% agreement in any weight category), fewer boys with overweight/obesity reported a good relationship with their PE teacher compared to their peers, while no such difference was observed in girls.

Consistent with the findings of the present study, PE has been shown to be a popular subject among adolescents in previous studies [25],[28]. Studies examining sex differences in attitudes towards and the perceived importance of PE, however, have yielded mixed results [25],[28],[37],[38]. It can, therefore, be argued that differences in the perception of PE between boys and girls may be attributed to the content of PE rather than differences in motivation towards PA in general. Available research indicates that boys prefer competitive team sports and challenging activities that include an element of risk, while girls tend to prefer less competitive fitness activities or dance that have a stronger social component [39],[40]. Biological and behavioral differences that generally result in greater strength and endurance, as well as better hand-eye coordination in boys, may further contribute to a higher perceived competence in PE among boys [41], which could contribute to their preference of competitive activities [42]. In addition, it should be considered that girls mature, on average, earlier than boys, and physiological and psychological changes along with alterations in body image may lead to shifts in attitudes towards PE [30]. Such differences need to be to be considered in the selection of activities during PE. Furthermore, girls reported that fitness activities are more likely to transfer into adulthood compared to traditional sports [39]. Fitness infusion interventions that include circuit training or high-intensity interval training, have also been associated with larger improvements in body composition [43], which may facilitate a sustained engagement in PA. A focus on health and fitness, rather than competitive sports, therefore, could provide more enjoyable experiences during PE among girls, which may lead to similar attitudes towards PE as observed in boys.

A similar argument can be made regarding the observed differences in attitudes towards and the perceived importance of PE across weight categories. While adolescents with overweight/obesity perceived PE to be less important than their peers, there was no difference in the perceived health benefits of PE across weight categories. Other studies also showed an inverse association between the preference for PE and body weight [28] or body fat and enjoyment of PE [44]. Specifically, bullying or disrespect may lead to a feeling of hostility in PE among youth with excess body weight [45]. Enjoyment of PA in general, however, did not differ between adolescents with excess body weight and their peers [46]. It can, therefore, be argued that inappropriate lesson planning and poor teaching strategies may be key contributors to poorer attitudes towards PE in adolescents with excess body weight rather than a lack of interest or motivation towards PA [47],[48]. Negative experiences stemming from a perceived lack of ability may contribute to negative perceptions of PE. A stronger emphasis on the mastery of personal goals rather than external competition and active engagement of the teacher, on the other hand, have been shown to increase motivation and excitement towards PE [28]. PE classes that focus on individual or team development rather than competition, along with outdoor activities, also have resulted in the highest PA levels [9]. Furthermore, high-intensity interval training has been shown to improve health-related physical fitness in overweight or obese youth [49]. As excess body weight has been associated with a lower participation in club sports [50],[51], PE may be the only setting for structured PA in adolescents with overweight/obesity. It is, therefore, crucial to provide positive experiences during PE [52],[53], which could encourage a more active lifestyle among this group.

Considering that an important aspect of PE is the promotion of an active lifestyle, it is concerning that the positive attitude towards PE has been consistently shown to decline with increasing age during adolescence [25],[29]. A potential reason for the decline could be the repetitive nature of activities performed, which may lead to a lack of challenge and boredom during PE [25]. Additionally, cognitive and socio-emotional development most likely contribute to changes in attitudes towards PE as self-competence and task values associated with PA have been shown to decline with increasing age during adolescence [54]. As adolescents mature, it becomes increasingly important to engage them in activities that provide personal meaning to facilitate sustainable PA participation. The integration of individual activities rather than traditional team sports along with diverse activities have been suggested to contribute to sustained interest in PE [25],[55]. As research has shown that adolescents engage in and participate in activities in which they feel competent, as well as those that are considered to be important [56],[57], it is important to emphasize the value of PA and foster a belief that success is possible if sufficient effort is provided. This will enhance attitudes towards PE as adolescents move closer towards adulthood [56]. A higher perceived competence has also been associated with greater participation in leisure time PA [42],[58], which can support a more active lifestyle later in life.

The importance of facilitating leisure time PA is further emphasized by the fact that quality-based PE interventions have been associated with greater benefits for health-related fitness and body composition compared to increasing the number of PE lessons [9],[43]. Specifically, the implementation of direct instruction teaching methods that focus on improving fundamental movement skills has been suggested as the most effective strategy to increase PA in youth [43]. This requires highly qualified teachers who are proficient in classroom organization and management. In addition, to sufficient activity time, constructive feedback and reflection appear to be critical, along with the establishment of consistent routines during PE classes [9]. Mastery-focused approaches in PE have also been shown to contribute to the development of physical literacy and lifelong physical activity skills that facilitate long-term enjoyment and PA independence [9],[59].

Some limitations of the present study, however, should be considered when interpreting the results of this study. Given the nature of the study, the data consist of self-reported information, which carries an inherent risk of bias and may limit objectivity. Body weight and height, on the other hand, were measured, allowing for a more accurate estimation of weight status than self-reported information. Further, no information was available on socioeconomic status, parental education, or the participants' physical activity level, all of which could affect attitudes towards PE. Engagement in PE or the content of PE was not assessed either, but attendance is mandatory in Austria and teachers are supposed to follow a standardized curriculum. It should also be considered that cross-sectional data were used in examining age related differences. Additional research using longitudinal data, along with the assessment of potential confounding variables, therefore, is needed to examine changes over time in the respective variables more clearly in order to enhance the understanding of the role of PE for the promotion of an active and healthy lifestyle. Despite these limitations, the large sample size and inclusion of both pre- and post-pubertal adolescents, however, provide valuable insights into adolescents' perceived importance of and attitudes towards PE, which can significantly influence their future life choices.

## 5. Conclusions

The findings of this study highlight the important role of PE in promoting an active lifestyle, as more than two-thirds of the participants reported that PE motivates them to engage in leisure time PA. In addition, PE is a popular subject among adolescents even though girls, as well as those with higher body weight and older age, have been shown to display a lower attitude towards PE and consider it to be less important than their peers. These groups, however, may be particularly dependent on PE to ensure regular PA, due to a lack of other opportunities [29]. It is, therefore, essential to provide quality PE that considers different motives for participation when designing PE curricula and to incorporate activities that engage all students. In addition, active teacher engagement and the facilitation of positive experiences by including elements of challenge and success, as well as joyful activities that contribute to the development of motor competence, appear to be critical to ensure positive attitudes towards PE and PA in general, which promote an active lifestyle.

## Use of AI tools declaration

The authors declare they have not used Artificial Intelligence (AI) tools in the creation of this article.
